# Selective Endocytosis-Mediated Omicron S1-RBD Internalization Revealed by Reconstitution of ACE2-S1-RBD Interaction on Micropatterned Membrane Substrates

**DOI:** 10.3390/ijms262010216

**Published:** 2025-10-21

**Authors:** Angelin M. Philip, S. M. Nasir Uddin, Zeyaul Islam, Prasanna R. Kolatkar, Kabir H. Biswas

**Affiliations:** 1 College of Health & Life Sciences, Hamad Bin Khalifa University, Doha, Qatar; angelinmaryphilip@gmail.com (A.M.P.);; 2 Qatar Biomedical Research Institute, Hamad Bin Khalifa University, Doha, Qatarpkolatkar@hbku.edu.qa (P.R.K.)

**Keywords:** ACE2, SARS-CoV-2, S1-RBD, supported lipid bilayer, endocytosis, cytoskeleton

## Abstract

The SARS-CoV-2 spike protein, through its receptor binding domain (S1-RBD), binds to the angiotensin-converting enzyme 2 (ACE2) receptor on the host cell membrane, leading to viral infection. Several mutations in S1-RBD in SARS-CoV-2 variants are known to enhance infection through an increased affinity for ACE2. While many reports are available describing the SARS-CoV-2 infection mechanism, there is a dearth of studies towards understanding the initial interaction of the S1-RBD with ACE2 on living host cells and the role of endocytosis and cytoskeleton in the process. Here, we reconstituted the interaction between S1-RBD- and ACE2-expressing host cells in a hybrid live cell-supported lipid bilayer (SLB) platform enabling live monitoring of the interaction between S1-RBD on SLBs and the ACE2 receptor on living cells and showed that cells depleted Omicron S1-RBD from SLB corrals, likely through endocytosis. Specifically, interaction of living host cells with S1-RBD-functionalized SLB substrates resulted in the enrichment of S1-RBD and ACE2 at the cell–SLB interface. Interaction of host cells with wild type (WT), Omicron, and Omicron Revertant S1-RBD functionalized on micron-scale SLB corrals, which mimic viral membranes but are flat, also resulted in their enrichment. However, cells interacting with Omicron S1-RBD revealed a depletion of the protein from many corrals, which was generally not observed with the WT S1-RBD and was reduced with the Omicron Revertant, which contains the Q493R mutation reversion, S1-RBD. Further, S1-RBD depletion coincided with the localization of the early endosomal marker EEA1. Importantly, treatment of cells with the clathrin inhibitor, pitstop 2, but not the myosin II inhibitor, blebbistatin, significantly reduced Omicron S1-RBD depletion. Collectively, these observations suggest that the SARS-CoV-2 Omicron variant has evolved, through mutations in its S1-RBD, to take advantage of the cellular endocytic pathway for enhanced infection, which is not observed with the parental SARS-CoV-2 and appears to be lost in the Omicron Revertant variant. Additionally, these results underscore the significance of the hybrid live cell–SLB platform in studying SARS-CoV-2 S1-RBD-ACE2 interaction and the potential impact of mutations in the S1-RBD on adapting to a specific cellular entry mechanism.

## 1. Introduction

The emergence of the SARS-CoV-2 virus, the causative agent of the COVID-19 pandemic, has led to extensive scientific investigations into various aspects of its infection pathway and, in particular, the interaction of its spike protein with host cell membrane-localized receptors [1,2]. The SARS-CoV-2 spike protein exists as a homotrimer on the viral envelope and comprises an S1 subunit, which contains the receptor binding domain (S1-RBD) that interacts with the angiotensin-converting enzyme 2 (ACE2) receptor expressed in host cell membranes, and an S2 subunit, which mediates the viral membrane fusion with the host cell membrane [3,4,5]. The ACE2-S1-RBD interaction is a key determinant of infectivity and transmissibility of the virus [6]. Unlike other structural proteins, such as the nucleocapsid (N), membrane (M), or envelope (E) proteins, which are involved in virion assembly and structural integrity, the S1-RBD directly governs host specificity, cell tropism, and the initial step of viral entry. Furthermore, it is the most variable region of the spike protein across emerging variants, with mutations frequently linked to changes in transmissibility, immune evasion, and vaccine escape. This functional and evolutionary significance makes the S1-RBD a critical focus for mechanistic studies aimed at understanding variant behavior and virus–host interactions at the molecular level. Further, mutations in the S1-RBD have the potential to increase the affinity of the protein for ACE2, as well as reduce the efficacy of vaccines and neutralizing antibodies against the virus [7,8,9,10]. Indeed, the parental (wild type; WT) SARS-CoV-2 has continuously evolved, giving rise to multiple variants with distinct genetic signatures [1]. Of these, the Omicron variant emerged with 37 mutations, particularly in the spike protein, and 15 of these are in S1-RBD. These mutations, such as G339D, K417N, S373P, S375F, T478K, N440K, S477N, E484A, Q498R, N501Y [11], Q493R [12], and Y505H, may impact the structure and affinity of the S1-RBD for ACE2, potentially impacting infectivity, transmissibility, and immune evasion [13,14].

Viral entry via viral–host cell interaction is a crucial step for SARS-CoV-2 infection and pathogenesis [15,16]. While initial interaction between the S1-RBD and ACE2 has been generally understood to result in the host–viral membrane fusion, leading to the release of the viral genetic material into the host cell, SARS-CoV-2 has also been reported to enter host cells via both canonical (clathrin-mediated) [17] as well as non-canonical (non-clathrin-mediated) endocytic pathways [18,19,20]. The clathrin-mediated endocytosis (CME) of SARS-CoV-2 involves the recruitment of adaptor proteins that connect the clathrin coat with the plasma membrane. Adaptor protein 2 (AP-2) is a key player in this process, facilitating the assembly of clathrin-coated pits and the concentration of cargo as they invaginate and pinch off to form clathrin-coated vesicles [21]. Furthermore, Early Endosome Antigen 1 (EEA1), a tethering protein that plays a crucial role in the fusion of early endosomes with the endocytic vesicles, facilitates the maturation of early endosomes [22]. Indeed, EEA1 is essential for the regulation of endosomal dynamics and cargo trafficking, contributing to the efficient internalization and intracellular transport of the virus following its entry via clathrin-mediated endocytosis [23,24]. Importantly, cell entry of the Omicron variant was not affected upon inhibition of Transmembrane Serine Protease 2 (TMPRSS2), as much as in the case of the WT or the Omicron Revertant variants [25], suggesting an altered mechanism of internalization utilized by the Omicron variant that likely serves as a basis for its enhanced infection of host cells.

Numerous studies have explored the infection mechanism of SARS-CoV-2 and the effects of mutations in the S1-RBD. However, there is limited research on the initial interaction between the S1-RBD and ACE2 in living cells and the impact of mutations in S1-RBD on this interaction. In this regard, the hybrid live cell-supported lipid bilayer (SLB) platform, wherein living cells expressing a receptor of interest interact with SLBs assembled on solid supports such as glass and functionalized with ligands of interest, can prove to be insightful. SLBs provide a native, cell membrane-like surface for reconstituting signaling systems by retaining lateral fluidity of the ligand of interest on a two-dimensional surface much like the cell membrane, and allow assembly of receptor–ligand complexes upon interaction with living cells into clusters, a process that has been shown to profoundly impact subsequent signaling events in the cell [26,27,28]. The hybrid live cell–SLB platform has been successfully utilized in dissecting mechanisms of several juxtracrine signaling systems, including the immunological synapse formed by the cytotoxic T-cell and an antigen-presenting cell [29,30], the signaling complex formed by the Eph (erythropoietin-producing human hepatocellular) family of receptors and the ephrin family of ligands [31], the integrin family of receptors [32], and the cadherin family of proteins [33]. Further, the fabrication of membrane microarrays such as circular corrals has been useful in spatially segregating SLB-functionalized ligands into individually addressable entities to control and monitor receptor–ligand complex clustering and cellular signaling in a spatially resolved fashion, which is non-trivial to achieve in typical viral infection studies involving living cells and viral particles [31,34,35,36].

In this manuscript, we reconstituted the ACE2-S1-RBD interaction in a hybrid live cell–SLB platform by functionalizing synthetic, planar SLBs with recombinantly purified S1-RBD protein and allowing ACE2-expressing A549 cells to interact with the SLB substrate, leading to the assembly of ACE2-S1-RBD clusters at the cell–SLB interface. Having successfully reconstituted the ACE2-S1-RBD interaction on planar SLB substrates, we then studied the differences in the interaction of the WT, Omicron, and Omicron Revertant S1-RBD on micropatterned SLB corral substrates generated using deep UV lithography. Cells interacting with S1-RBD corrals also showed clustering of the proteins as observed with the planar SLB substrates. However, interaction of the cells resulted in a depletion of the Omicron S1-RBD from SLB corrals, which was either not observed or was minimal in the case of the WT or the Omicron Revertant S1-RBD-functionalized SLB corrals. Treatment of cells with pitstop 2, an endocytosis inhibitor, resulted in a significant reduction in Omicron S1-RBD depletion from SLB corrals, suggesting a role for clathrin-mediated endocytosis in Omicron S1-RBD depletion. Importantly, early endosomal marker protein, EEA1, showed a higher colocalization with Omicron S1-RBD-functionalized SLB corrals. These results point towards the potentially increased adoption of the endocytosis-mediated infection pathway by the Omicron variant vis-à-vis the WT and Omicron Revertant variant.

## 2. Results

### 2.1. Reconstitution of ACE2-S1-RBD Interaction in a Hybrid Live Cell–SLB Platform

**Figure 1:**
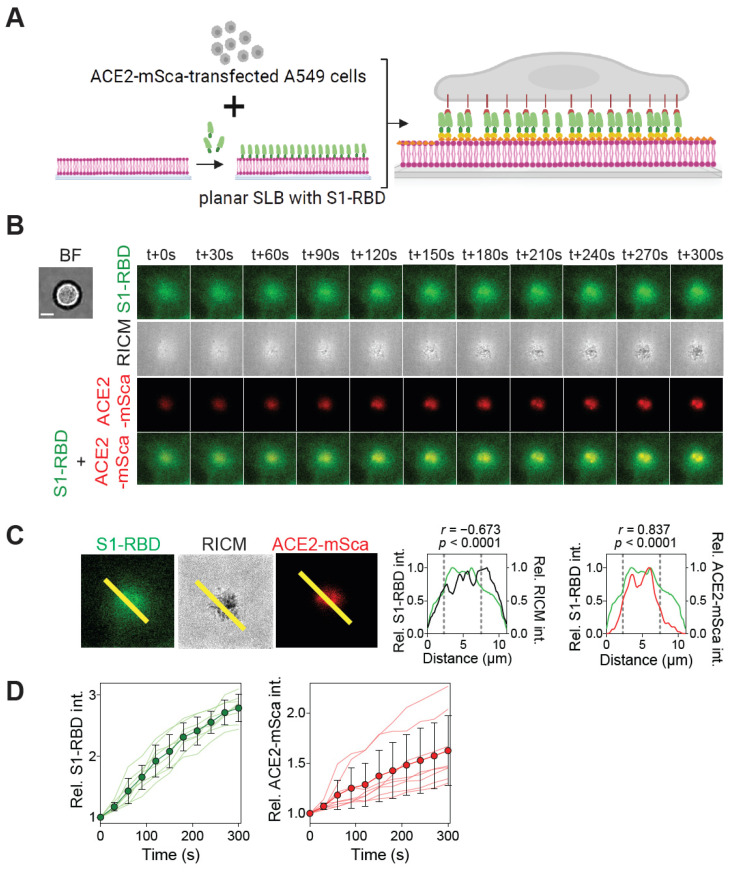
Reconstitution of the ACE2-S1-RBD interaction between living cells and SLB substrate. (**A**) Schematic showing reconstitution of the ACE2-S1-RBD interaction in a hybrid live cell–SLB format. (**B**) BF, TIRF (TIRF 488 for S1-RBD and TIRF 561 for ACE2), and RICM time-lapse images of A549 cells transfected with ACE2-mSca (red) interacting with the S1-RBD (green), which is immobilized on the supported lipid bilayer (SLB) via a poly-His tag–Ni-NTA interaction. The S1-RBD carries a 12X poly-His tag (yellow), while the lipid bilayer (magenta) is functionalized with Ni-NTA lipids (orange). Note the enrichment of the protein after t + 90 s (30 min post cell–SLB interaction) and colocalization of ACE2-mSca and S1-RBD fluorescence signal (yellow) at the cell–bilayer interface. (**C**) Left panel: TIRF (TIRF 488 for S1-RBD and TIRF 561 for ACE2) and RICM images of ACE2-mSca-expressing A549 cells interacting with S1-RBD SLB substrate. Lines (yellow) used for intensity correlation analysis. Right panel: Graphs showing intensity profiles of S1-RBD and RICM and S1-RBD and ACE2-mSca. Note the inverse correlation between S1-RBD and RICM and a positive correlation between S1-RBD and ACE2 in the region highlighted with gray dotted lines. (**D**) Graphs showing relative intensities of S1-RBD and ACE2-mSca at the cell–bilayer interface over time. Data shown mean ± s.d. and data from individual cells are shown as line traces. *n* = 3 biological replicates, 25 cells each. Scale bar, 5 µm.

To monitor the initial interaction of SARS-CoV-2 S1-RBD with the ACE2 receptor expressed in living cells, we reconstituted the interaction in a hybrid live cell–SLB format [30,31,34,35,37]. For this, SLBs were assembled on acid-cleaned coverslips and then functionalized with recombinantly purified S1-RBD tagged with mGreenLantern (mGL) fluorescent protein through the Ni-NTA-poly-His tag interaction [35]. A549 epithelial cells transiently expressing ACE2 receptor tagged with the mScarlet fluorescent protein (ACE2-mSca) were then seeded onto the SLB substrate functionalized with S1-RBD, and the cells were imaged for interaction with the SLB substrate (Figure 1A). Time-lapse Total Internal Reflection Fluorescence (TIRF) microscopy imaging revealed enrichment of S1-RBD on SLBs in contact with the cells, as ascertained from Reflection Interference Contrast Microscopy (RICM) imaging (Figure 1B). Time-lapse RICM imaging reveals progressive cell attachment, visualized as an increase in the dark regions within the RICM channel over time. Although A549 cells typically adopt a spread morphology on rigid substrates, in our experiments with the SLB substrates, the cells maintained a predominantly circular shape even over extended imaging periods. This is a common feature of cells interacting with fluid SLB substrates that has been reported previously in numerous previous studies [29,30,34,35,38,39,40,41,42]. Simultaneous TIRF imaging in the red channel for ACE2-mSca revealed enrichment of ACE2 on the cell membrane in the form of clusters (Figure 1B), suggesting the interaction of cell membrane-localized ACE2-mSca with S1-RBD on SLB (Figure 1B). Further, comparison of S1-RBD fluorescence and RICM intensity line scans showed a negative correlation (Pearson correlation, *r* = −0.673, *p* < 0.0001) with a decrease in RICM intensity at regions of S1-RBD enrichment (Figure 1C), suggesting close contact between the cell membrane and the SLB substrate. In contrast, a comparison of S1-RBD and ACE2-mSca fluorescence showed a strong positive correlation (*r* = 0.87, *p* < 0.0001) (Figure 1C), consistent with S1-RBD binding specifically to ACE2 expressed on the host cell membrane. Using time-lapse imaging, we quantified the time-dependent changes in the relative intensities of S1-RBD and ACE2-mSca at the cell–SLB interface, which showed a gradual increase over time (Figure 1D), indicating their progressive accumulation at the interface. Together, these results show that SARS-CoV-2 S1-RBD functionalized on SLBs binds to the ACE2 receptor expressed on the host cell membrane, and thus, we have established a platform to probe the interaction of S1-RBD from SARS-CoV-2 variants and the role of cellular processes such as endocytosis or the cytoskeleton.

### 2.2. Significant Depletion of Omicron S1-RBD- from SLB Corral upon Interaction with A549 Cells

**Figure 2:**
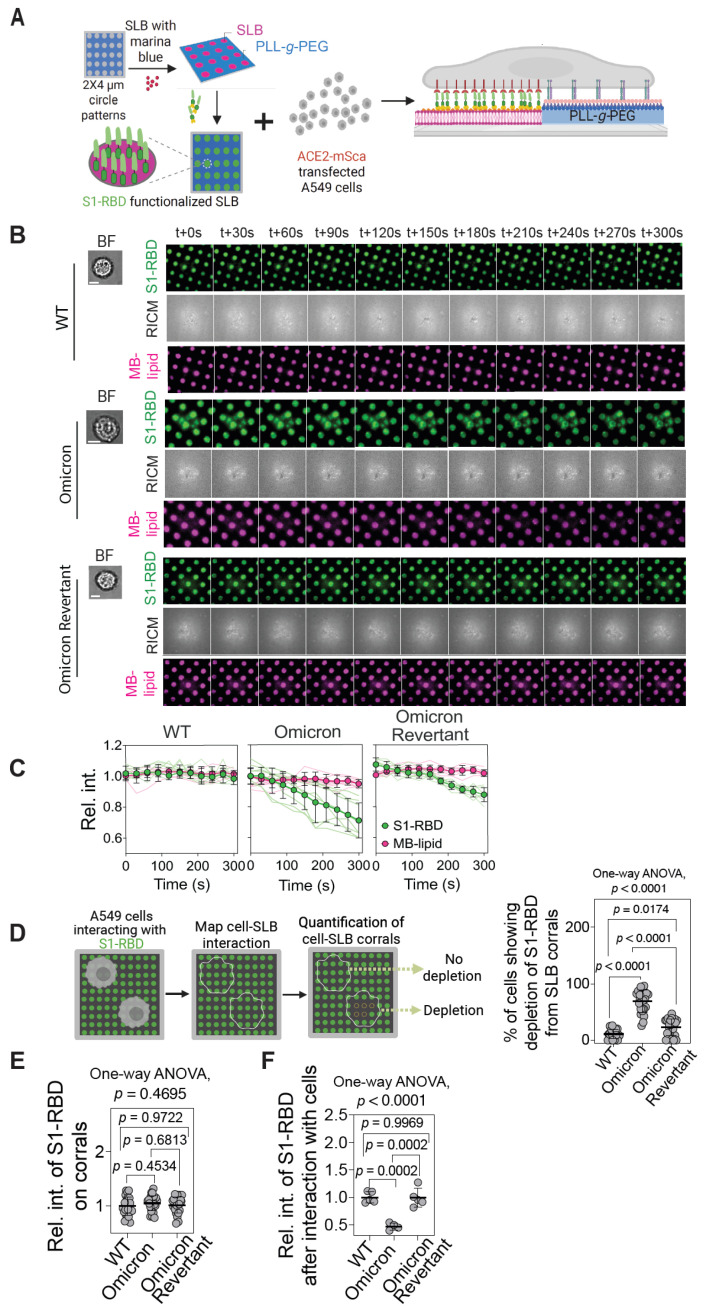
Selective depletion of Omicron S1-RBD from SLB corrals upon interaction with ACE2-expressing cells. (**A**) Schematic showing the reconstitution of the ACE2-S1-RBD interaction between ACE2-expressing A549 cells and S1-RBD-functionalized micropatterned SLB substrate. (**B**) BF, TIRF 488 (S1-RBD), RICM, and TIRF 405 (MB-lipid) time-lapse images of A549 cells interacting with either WT or Omicron or Omicron Revertant S1-RBD-functionalized SLB corrals. Note the decrease in Omicron S1-RBD intensity at t + 90 s. (**C**) Graphs showing relative intensities of WT, Omicron, and Omicron Revertant S1-RBD-functionalized SLB. Note the decrease in Omicron S1-RBD intensity, while lipid intensity remains unchanged. Data show mean ± s.d. and data from individual cells are shown as line traces. *n* = 3 biological replicates, 25 cells each. (**D**) Schematic (left) illustrating the quantification of the percentage of cells showing S1-RBD depletion, and graph (right) showing percentage of cells showing depletion of S1-RBD fluorescence intensity from SLB corrals after 30 min of incubation of the cells with the micropatterned substrates. Data shown are mean ± s.d. and *p*-values shown were obtained from one-way ANOVA analysis; adjusted *p*-values have been shown for comparison between groups; *n* = 3 biological replicates, 25 frames each. (**E**,**F**) Graph showing relative fluorescence intensity of WT, Omicron, and Omicron Revertant S1-RBD proteins functionalized on micropatterned substrates prior to cell incubation (**E**) and after interaction with cells. Data shown are mean ± s.d. and *p*-value shown were obtained from one-way ANOVA analysis; adjusted *p*-values have been shown for comparison between groups; *n* = 3 biological replicates, 25 frames each in (**E**) and 4–5 corrals in (**F**). Scale bar, 5 μm.

Having established the ACE2-S1-RBD interaction in a hybrid live cell–SLB format, we attempted to utilize micropatterned SLB corral substrates [30,31,34,37,43,44] to spatially restrict the assembly of ACE2-S1-RBD complexes using either WT, Omicron, or Omicron Revertant S1-RBD with ACE2 (Figure 2A). For this, acid-cleaned coverslips were coated with PLL-*g*-PEG-biotin, and the polymer was etched using a photomask with circular features of 2 μm in diameter and a spacing of 4 μm under deep-UV illumination. Following etching, SLB corrals were assembled through the vesicle fusion method, and the corrals were functionalized with either the WT or the Omicron or the Omicron Revertant S1-RBD. Single-cell suspension of A549 epithelial cells transfected with ACE2-mSca was then seeded on the micropatterned SLB corral substrates [30,31,37] and their interaction was monitored through microscopy.

Fluorescence and RICM imaging revealed that the ACE2-mSca-expressing cells interacted with all three types (WT, Omicron, and Omicron Revertant S1-RBD functionalized) of micropatterned SLB corral substrates (Figure 2B). Time-lapse fluorescence imaging revealed that WT S1-RBD-functionalized SLB corrals retained fluorescence over time. In contrast, Omicron S1-RBD corrals exhibited a substantial decrease in fluorescence intensity (green) (Figure 2B), suggesting significant protein depletion over time. The Omicron Revertant showed an intermediate behavior, with significantly reduced fluorescence loss compared to Omicron but more than WT (Figure 2B). RICM imaging showed cell attachment to the substrate over time in all three functionalized SLB substrates with either WT, Omicron, or Omicron Revertant, indicating consistent cell–substrate interactions. Notably, only the S1-RBD fluorescence intensity (green) varied between different S1-RBD-functionalized SLB corrals, while the lipid fluorescence intensity (magenta) remained stable throughout, confirming structural integrity of the SLB (Figure 2B). Quantitative analysis of fluorescence microscopy images further supported these observations (Figure 2C), in that the fluorescence intensity of Omicron S1-RBD gradually decreased over time, whereas the fluorescence intensity of WT and Omicron Revertant S1-RBD remained stable over time (Figure 2C). We further manually quantified the percentage of corrals interacting with cells, showing a decrease in S1-RBD fluorescence intensity. We observed a significantly higher proportion of Omicron S1-RBD-functionalized SLB corrals exhibiting S1-RBD depletion compared to Omicron Revertant and WT (69 ± 7.6, 23 ± 5.7, and 11 ± 8.2% for Omicron, Omicron Revertant, and WT, respectively) (Figure 2D). To rule out the possibility of a difference in the molecular density of the S1-RBD proteins on the SLB corrals, we quantified the fluorescence intensity of the S1-RBD protein prior to interaction with cells. This revealed similar densities of the WT, Omicron, and Omicron Revertant S1-RBD proteins on the SLB corrals (Figure 2E), indicating that the differences in the percentage of cells showing depletion of S1-RBD between different substrates are not due to a difference in molecular density of the proteins on the SLB corrals. On the other hand, comparison of S1-RBD fluorescence intensity in corrals that are not interacting with cells and those that are interacting with cells showed a significant reduction in the case of Omicron S1-RBD (Figure 2F). These results suggest an enhanced interaction of the Omicron S1-RBD, as compared to the WT or Omicron Revertant S1-RBD, with ACE2 likely due to its higher affinity for the receptor, potentially resulting in its internalization into the cells.

### 2.3. Omicron S1-RBD Depletion from SLB Corrals Is Mediated by the Host Cell Endocytic Machinery

Following the observation of selective depletion of Omicron S1-RBD from SLB corrals, we attempted to delineate the mechanism underlying the phenomenon. In this regard, we focused on the host cell endocytic machinery. Specifically, we monitored the localization and dynamics of the EEA1 protein, which is involved in the sorting of the endosome system. More specifically, EEA1 is involved in the docking and fusion of endocytic vesicles with early endosomes. It has been implicated in the life cycle of various pathogens, including the SARS-CoV-2 virus [17,20,21,22,45]. While there are a number of proteins that can serve as an endosomal marker, we decided to utilize EEA1 as it labels the early endosome, indicating entry of the cargo into the endosome, and has been widely validated.

**Figure 3:**
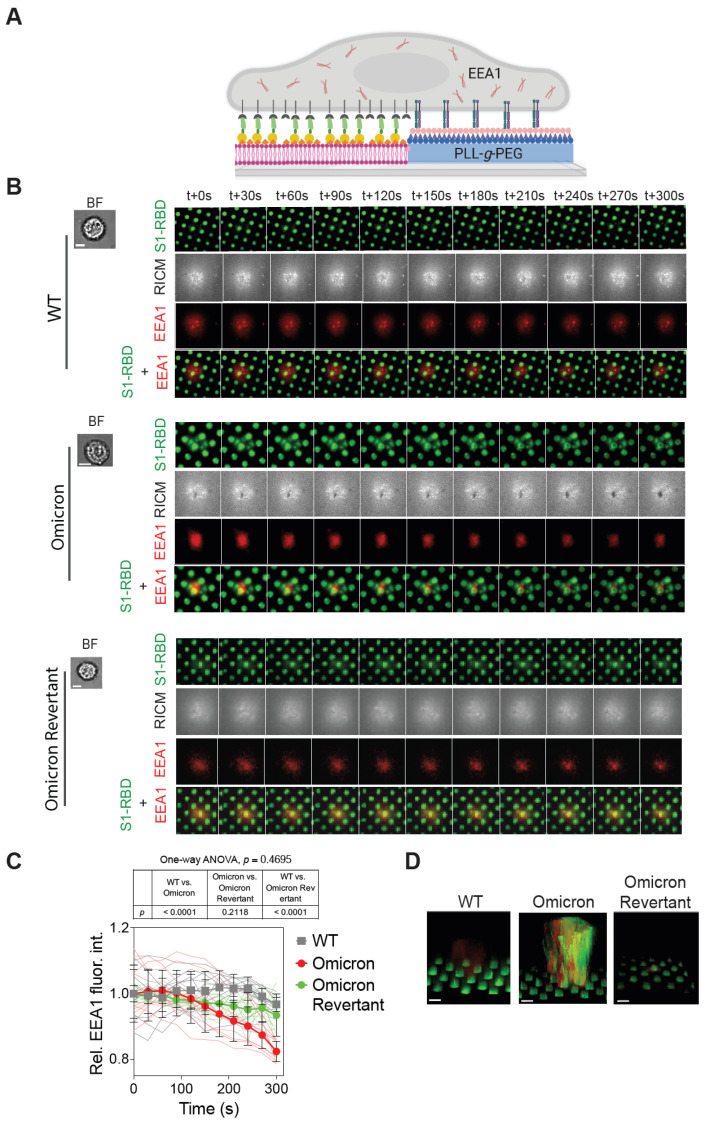
Depletion of Omicron S1-RBD from SBL corrals is associated with a decrease in EEA1 intensity. (**A**) Schematic showing interaction of EEA1-expressing A549 cells interacting with either WT or Omicron or Omicron Revertant S1-RBD-functionalized SLB corrals. (**B**) BF, TIRF (TIRF 488 for S1-RBD and TIRF 561 for EEA1), and RICM time-lapse images of A549 cells transfected with EEA1 (red) interacting with either WT or Omicron or Omicron Revertant S1-RBD (green) functionalized micropatterned substrates. (**C**) Graph showing relative EEA1 fluorescence intensity at the cell–bilayer interface in A549 cells interacting with the indicated S1-RBD-functionalized micropatterned substrates over time. Data shown are mean ± s.d. and *p*-values shown were obtained from one-way ANOVA analysis at 300 s; adjusted *p*-values have been shown for comparison between groups; *n* = 3 biological replicates, 25 frames each. (**D**) Images showing 3D volume reconstruction of confocal z-scan images of EEA1-expressing A549 cells interacting with the indicated S1-RBD-functionalized micropatterned substrates. Note the green (S1-RBD) and red (EEA1) fluorescence intensities in the case of Omicron S1-RBD functionalized substrate. Scale bar, 5 μm.

To achieve this, we allowed A549 cells transfected with an EEA1 fused with the red fluorescent protein (EEA1-RFP) to interact with micropatterned SLB substrates [30,31,34,37,43,44] functionalized with either WT or Omicron or Omicron Revertant S1-RBD (Figure 3A). Time-lapse TIRF and RICM imaging of cells interacting with micropatterned SLB substrates showed minimal enrichment and clustering of EEA1-RFP at cell–SLB contact sites in WT S1-RBD-functionalized corrals, indicating limited recruitment of early endocytic machinery (Figure 3B). In contrast, Omicron S1-RBD substrates exhibited a noticeable EEA1-RFP clustering at these contact regions (Figure 3B), suggesting the possibility of an active involvement of endocytic pathways in ACE2–S1-RBD interactions. On the other hand, while cells interacting with the Omicron Revertant S1-RBD-functionalized SLB corrals showed some EEA1 enrichment, it was significantly less compared to the cells interacting with Omicron S1-RBD-functionalized SLB corrals (Figure 3B). Importantly, S1-RBD depletion correlated with a reduction in EEA1 fluorescence intensity over time, with EEA1–S1-RBD colocalization. This phenomenon was most evident in the Omicron S1-RBD condition, where a loss of EEA1 signal coincided with increased S1-RBD depletion, suggesting a possible active internalization via the endocytic pathway. In contrast, although some EEA1 localization was observed with the Omicron Revertant, the intensity of EEA1–S1-RBD colocalization remained relatively stable over time, indicating limited or slower internalization. Quantitative analysis was performed to assess the time-dependent changes in EEA1 fluorescence intensity in response to interactions with S1-RBD. This analysis showed a time-dependent decrease in EEA1-RFP intensity on Omicron S1-RBD-functionalized substrates after 30 min of cell–SLB corral interaction. In contrast, no significant reduction was observed on substrates functionalized with WT or Omicron Revertant S1-RBD. The mean EEA1 intensity for Omicron was 0.94 ± 0.10, and for Omicron Revertant, 0.92 ± 0.09; (*p* < 0.0001 for WT vs. Omicron, *p* = 0.2118 for WT vs. Omicron Revertant, and *p* < 0.0001 for Omicron vs. Omicron Revertant) (Figure 3C). These observations indicate a possible internalization of Omicron S1-RBD by the cell, as EEA1 assembles only during endocytosis, and is known to dissipate during the late endocytic phase [22]. To investigate this further, we performed *z*-scan confocal imaging of cells interacting with either WT, Omicron, or Omicron Revertant S1-RBD-functionalized micropatterned substrates. Three-dimensional (3D) volume reconstruction from *z*-scan images revealed distinct patterns of fluorescence intensity in cells interacting with different S1-RBD substrates. In cells interacting with Omicron S1-RBD substrates, both EEA1-RFP (red) and S1-RBD (green) fluorescence intensities were observed inside cells, whereas only EEA1-RFP fluorescence was detected when interacting with the WT S1-RBD substrate. Furthermore, *z*-scan images of cells interacting with Omicron Revertant S1-RBD-functionalized substrates showed colocalization and interaction of EEA1-RFP (red) fluorescence at the corrals, but no internalization of the S1-RBD was detected (Figure 3D). Together, these results indicate that an enhanced interaction between the Omicron S1-RBD and ACE2 receptor likely results in endocytosis-mediated internalization of the protein from the SLB corral into the cell.

### 2.4. Pitstop 2 Abrogates, While Blebbistatin Partially Reduces, Omicron S1-RBD Depletion from SLBs

To further assess the role of endocytosis in the depletion of Omicron S1-RBD from SLB corrals, we took advantage of the chemical inhibitors, pitstop 2 [46], which specifically targets clathrin and hinders the assembly of clathrin-coated pits by impeding the functionality of the AP2U2 protein at the membrane [17,47], offering the possibility of providing valuable insights into the mechanistic intricacies of endocytosis. Additionally, we used blebbistatin, the myosin II inhibitor, to assess the role of actomyosin tension [48]. We utilized micropatterned SLB substrates functionalized with either Omicron or Omicron Revertant S1-RBD for these experiments since cells interacting with WT S1-RBD-functionalized SLB corrals did not show significant internalization. Further, cells were transfected with either AP2U2-RFP (to understand the effect of pitstop 2) or Lifeact-mKate2 (to analyze the effect of blebbistatin on the actomyosin interaction) and pretreated with the inhibitors prior to seeding them on SLB corral substrates.

**Figure 4:**
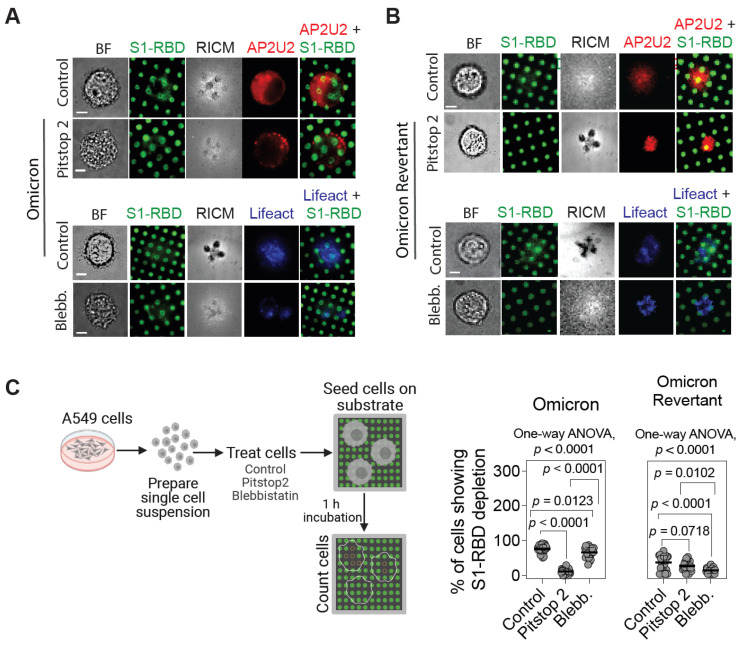
Reduced Omicron S1-RBD corral depletion with endocytic inhibitors. (**A**) Top panel: BF, TIRF (488 for S1-RBD and 561 for AP2U2), and RICM images of control and pitstop 2-treated A549 cells on Omicron S1-RBD-functionalized micropatterned SLB substrate showing a reduced localization of the AP2U2 at the cell–SLB interface and Omicron S1-RBD depletion from SLB corrals in pitstop 2-treated cells. Bottom panel: BF, TIRF (488 for S1-RBD and 561 for Lifeact-mKate2), and RICM of control and blebbistatin (Blebb.)-treated A549 cells on Omicron S1-RBD-functionalized micropatterned SLB substrate showing no significant effect on the depletion of Omicron S1-RBD from SLB corrals. (**B**) Top panel: BF, TIRF (488 for S1-RBD and 561 for AP2U2), and RICM images of control and pitstop 2-treated A549 cells on Omicron Revertant S1-RBD-functionalized micropatterned SLB substrate showing no significant effect on AP2U2 localization at the cell–bilayer interface and Omicron Revertant S1-RBD depletion from SLB corrals in pitstop 2-treated cells. Bottom panel: BF, TIRF (488 for S1-RBD and 561 for Lifeact-mKate2), and RICM images of control and blebbistatin (Blebb.)-treated A549 cells on Omicron Revertant S1-RBD-functionalized micropatterned SLB substrate showing a reduction in the depletion of Omicron Revertant S1-RBD from SLB corrals in blebbistatin-treated cells. Note that the cells were pre-treated with 50 μM of the inhibitors (pitstop 2 or blebbistatin) for 15 min, following which they were seeded on the micropatterned substrates and allowed to interact with the Omicron or Omicron Revertant S1-RBD for 1 h. Cells were fixed and imaged after 1 h incubation on the micropatterned substrates. (**C**) Left: schematic illustrating analysis of S1-RBD depletion in Omicron and Omicron Revertant SLB corrals upon inhibitor treatment. Right, graphs showing percentage of cells showing either Omicron (left) or Omicron Revertant (right) S1-RBD fluorescence intensity decrease from SLB corrals. Note the significant reduction in Omicron S1-RBD depletion in pitstop 2-treated cells and Omicron Revertant S1-RBD depletion in blebbistatin-treated cells. Data shown are mean ± s.d. and *p*-value shown were obtained from one-way ANOVA analysis; adjusted *p*-values have been shown for comparison between groups; *n* = 1 biological replicate; statistics based on randomized technical replicates. Scale bar, 5 µm.

Cells transfected with AP2U2 but not treated with pitstop 2 (control cells) interacting with Omicron S1-RBD substrates showed localization of AP2U2 on the membrane, whereas pitstop 2-treated cells showed reduced AP2U2 localization (Figure 4A, top panel). This suggests that pitstop 2 abrogated AP2U2 localization, impairing clathrin-mediated endocytosis, and thus may reduce Omicron S1-RBD depletion from SLB corrals. On the other hand, cells transfected with Lifeact-mKate2 and treated with blebbistatin showed a decrease in actin filaments at the cell–SLB interface, as compared to non-treated, control cells (Figure 4A, bottom panel). Importantly, RICM imaging cells treated with blebbistatin and interacting with Omicron S1-RBD-functionalized SLBs corral substrates revealed reduced cell–SLB contact, as compared to non-treated, control cells. However, blebbistatin treatment did not affect Omicron S1-RBD depletion from SLB corrals, as much as pitstop 2 treatment did. Similarly, AP2U2-transfected control cells interacting with Omicron Revertant S1-RBD substrates exhibited lesser AP2U2 localization at the cell–SLB interface, as compared to the cells interacting with Omicron S1-RBD substrates, and treatment with pitstop 2 did not show any further reduction in the AP2U2 localization (Figure 4B, top panel). As seen with cells interacting with Omicron S1-RBD substrates, blebbistatin treatment of cells interacting with Omicron Revertant S1-RBD substrates also showed decreased cell–SLB contact, as ascertained from RICM imaging (Figure 4B, bottom panel). Additionally, blebbistatin treatment further reduced the small amount of Omicron Revertant S1-RBD from SLB corrals (Figure 4B, bottom panel). We further quantified the percentage of cells interacting with the corrals showing S1-RBD depletion. In control cells, the S1-RBD depletion was significantly higher compared to pitstop 2-treated cells (75.9 ± 10.3 and 10.7 ± 3.1% for control and pitstop 2-treated cells, respectively; *p* < 0.0001), indicating a strong inhibitory effect of pitstop 2 on S1-RBD depletion, whereas treatment with the blebbistatin did not show a drastic reduction as seen with pitstop 2; rather, a modest yet significant reduction in Omicron S1-RBD depletion (75.9 ± 10.3 and 66.3 ± 14% for control and blebbistatin-treated cells, respectively; *p* = 0.0123) (Figure 4C, graph on left) was observed. Similarly, control and pitstop 2-treated cells did not exhibit a statistically significant difference in Omicron Revertant S1-RBD depletion (23.8 ± 6.7 and 16.2 ± 9.5% for control and pitstop 2-treated cells, respectively; *p* = 0.0718). However, in comparison to the control, blebbistatin-treated cells showed a reduction in Omicron Revertant S1-RBD depletion (23.8 ± 6.7 and 6.9 ± 2.7% for control and blebbistatin-treated cells, respectively; *p* < 0.0001 (Figure 4C, graph on right). These observations suggest that inhibiting clathrin-mediated endocytosis led to a significant reduction in Omicron S1-RBD depletion, while reducing actomyosin tension affected the depletion of Omicron Revertant S1-RBD. This implies that the interaction between Omicron S1-RBD and ACE2 may enhance endocytosis, potentially increasing Omicron’s spread and transmission. In contrast, the Omicron Revertant S1-RBD with the R493Q reversion likely relies more on cytoskeletal dynamics for entry, showing reduced use of the endocytic pathway [49]. While studies directly linking the R493Q mutation to cytoskeletal remodeling are limited, it is known that viral infections often induce cytoskeletal changes to facilitate entry and replication [50]. The spike protein mutations, including R493Q, can alter interactions with host cell receptors and potentially influence cytoskeletal dynamics indirectly through signaling pathways activated during infection [51,52].

## 3. Discussion

The emergence of the Omicron variant marked a significant increase in SARS-CoV-2 infectivity and transmission. While this could be largely attributed to an increased affinity of the spike protein for ACE2, we attempted to understand the impact of Omicron mutations on the initial interaction between membrane-bound S1-RBD protein and cell membrane localized ACE2 receptor through reconstitution of the system in a hybrid live cell–SLB platform. Our findings revealed the striking observation of depletion of Omicron S1-RBD from SLB corrals upon interaction with ACE2-expressing cells, in contrast to minimal depletion observed with the WT and Omicron Revertant S1-RBD. This suggests that Omicron S1-RBD is more readily internalized from the cell–SLB interface. Notably, Omicron S1-RBD exhibited increased colocalization with the early endosomal marker, and this depletion was significantly reduced upon treatment with pitstop 2, a commonly used clathrin-mediated endocytosis inhibitor. These observations strongly implicate clathrin-dependent endocytosis as a key mechanism driving Omicron S1-RBD internalization. However, as pitstop 2 is known to exhibit off-target effects unrelated to clathrin function, these findings should be interpreted with caution. Further validation using complementary approaches will be important to confirm the specific involvement of clathrin-mediated pathways. Z-stack imaging further supported this conclusion by demonstrating intracellular localization of Omicron S1-RBD, a feature absent in WT and largely reduced in the Omicron Revertant S1-RBD. These results align with existing evidence suggesting that Omicron preferentially utilizes endocytic pathways for cell entry, as opposed to the TMPRSS2-mediated plasma membrane fusion favored by earlier variants. The reduced replication of the Omicron variant in TMPRSS2-rich tissues such as the lungs and intestines, and the diminished ability of the Omicron variant to form syncytia, are consistent with this altered entry mechanism and may contribute to differences in disease severity and pathogenesis [25]. Moreover, multiple studies have suggested that Omicron S1-RBD exhibits an increased binding affinity (5.5 ± 1.4 nM) with ACE2, whereas the Omicron Revertant, bearing a R493Q mutation reversal, further increases the binding affinity (2.4 ± 1.1 nM), as compared to the WT (27.5 ± 4.8 nM) [53,54,55]. While our findings support reduced reliance on TMPRSS2, several studies have demonstrated that the Omicron variant can still utilize TMPRSS2-mediated entry to a certain extent. For example, Shiliaev et al. (2022) showed that the Omicron variant retains the ability to replicate in human airway epithelial cells that express TMPRSS2, indicating that this protease-dependent entry pathway remains partially functional in relevant primary tissues [56]. Similarly, Zhao et al. (2022) observed that while fusion activity of the Omicron variant in TMPRSS2-expressing cells is reduced compared to the Delta variant, it is nonetheless detectable, suggesting that the variant does not completely abandon TMPRSS2-dependent entry [57]. Moreover, Saito et al. (2022) provided comparative analyses highlighting that the fusogenicity of the Omicron variant and pathogenicity are attenuated but not abolished, implying residual TMPRSS2 engagement [58]. These studies collectively underscore the flexibility of the entry mechanism of the Omicron variant, which appears to balance between endosomal uptake and TMPRSS2-dependent plasma membrane fusion in a cell type- and context-dependent manner. This context-dependent usage highlights the flexibility of the entry strategy of the Omicron variant and underscores the limitations of our model, which uses monomeric RBD and ACE2-overexpressing cells. Additionally, findings from steered molecular dynamic studies indicate that the increased affinity and greater number of interactions at the Omicron S1-RBD interface result in a higher force being necessary to pull away, as compared to the WT [53,59]. The reversion from R493Q in the Omicron Revertant restores some binding affinity to ACE2 and may sensitize variants to neutralization by certain monoclonal antibodies [60,61]. However, the Q493R mutation appears to confer an advantage by enhancing the ability of the virus to infect host cells [60,62]. This enhanced infectivity plays a significant role in facilitating the internalization process, as the virus can more effectively engage with and enter the host cell due to the stronger and more numerous interactions with its receptor [49].

## 4. Materials and Methods

### 4.1. Plasmid Design

The nucleotide sequences of WT and Omicron SARS-CoV-2 S1-RBD were obtained from GSAID (https://www.gisaid.org/) (accessed on 1 January 2022). The WT S1-RBD spanning residues from T333 to G526 of the S1-RBD were synthesized and subcloned into the pBAD33 plasmid to generate the pBAD33-WT-S1-RBD-mGL-His_12_ (WT S1-RBD; GenScript, Singapore) for bacterial expression and purification. Similarly, the nucleotide sequences obtained for Omicron were synthesized and subcloned into the pBAD33 plasmid to generate the pBAD33-Omicron-S1-RBD-mGL-His_12_ plasmid (Omicron S1-RBD). Omicron Revertant S1-RBD expression plasmid was generated through mutagenesis PCR using the pBAD33-Omicron-S1-RBD-mGL-His_12_ plasmid as the template and forward (5′-GCTATTTTCCGCTGCAGAGCTACAGCTTCCG-3′) and reverse (3′-CGCCTTCAAACTTCACTTCCGC-5′) primers designed to introduce the R493Q mutation. All protein constructs contained mGreenLantern (mGL) [63,64] fluorescent protein and His_12_-tag at the C-terminal (Supplementary Text S1).

### 4.2. Protein Expression and Purification

The S1-RBD expression plasmids, either pBAD33-WT-S1-RBD-mGL-His_12_, pBAD33-Omicron-S1-RBD-mGL-His_12_ or pBAD33-Omicron Revertant-S1-RBD-mGL-His_12_, were transformed into *Escherichia coli* BL21 (DE3) competent cells. A single colony of *E. coli* BL21 (DE3) strains carrying the plasmids was inoculated in 100 mL of Luria–Bertani (LB) medium supplemented with 50 μg/mL of chloramphenicol. An aliquot (1%) of the overnight-grown culture was inoculated in fresh LB medium supplemented with the same antibiotics and left shaking at 200 rpm for 2.5 h at 37 °C (OD_600_ was ~0.40–0.42). The protein expressions were induced with 0.2% L-Arabinose. The cultures were further incubated at 37 °C, 200 rpm for 3 h. The cells were harvested by centrifuging the culture at 8000 rpm for 15 min at 4 °C. The cell pellet was resuspended in lysis buffer (50 mM Tris-HCl, pH 8.2, 500 mM NaCl, 10% glycerol, 5 mM β-mercaptoethanol, and 20 mM imidazole) along with 0.2 mL of a mixture of protease inhibitors. Cell lysis was performed chemically, by incubation with hen egg white lysozyme, DNase I, and deoxycholate for 1 h in the cold room with gentle rotation. The soluble and insoluble cell fractions were separated by centrifuging the cell lysate at 18,000 rpm for 1 h at 4 °C. The supernatant was loaded onto an immobilized metal affinity column (nickel-nitrilotriacetic acid column) that was pre-equilibrated with equilibration buffer (50 mM Tris-HCl, pH 8.2, 500 mM NaCl, 10% glycerol, 5 mM β-mercaptoethanol, and 20 mM imidazole). The column was washed with 25 column volumes of wash buffer (50 mM Tris-HCl, pH 8.2, 500 mM NaCl, 10% glycerol, 5 mM β-mercaptoethanol, and 40 mM imidazole) to remove any non-specifically bound proteins. Finally, the bound protein was eluted with elution buffer (50 mM Tris-HCl, pH 8.2, 500 mM NaCl, 5% glycerol, 5 mM β-mercaptoethanol, and 300 mM imidazole) and subsequently dialyzed overnight against 50 mM Tris-HCl, pH 8.2, 150 mM NaCl, 5% glycerol, 5 mM β-mercaptoethanol. The proteins were concentrated and stored at −80 °C till further use. Further, we performed SDS-PAGE analysis to confirm the purity of WT, Omicron, and Omicron Revertant S1-RBD proteins (~58 kDa) (Supplementary Figure S1).

### 4.3. Supported Lipid Bilayer Preparation

Lipid 1,2-dioleoyl*sn*-glycero-3-phosphocholine (DOPC) was mixed in chloroform with 4 mole% of 1,2-dioleoyl-*sn*-glycero-3-[(N-(5-amino-1-carboxypentyl) iminodiacetic acid) succinyl] nickel salt (Ni-NTA-DOGS) (Avanti Polar Lipids, Alabaster, AL, USA) in a round-bottom flask. To visualize lipid bilayer fluidity, Marina Blue 1,2-dihexadecanoyl-*sn*-glycero-3-phosphoethanolamine (Marina Blue DHPE) (Life Technologies, Carlsbad, CA) was added to some vesicle preparations at a concentration of 1 mol% [31,32,34,35,36,38,44,65,66,67,68,69]. Chloroform was evaporated in a rotary evaporator under vacuum to leave a thin lipid film. To pattern the SLBs and prevent non-specific cell adhesion, glass substrates were pre-treated with poly(L-lysine)-graft-poly (ethylene glycol)-biotin (PLL-PEG-Biotin) (SuSoS AG, Dübendorf, Switzerland). PLL electrostatically adheres to the negatively charged glass surface, while PEG provides a passivating layer that reduces non-specific protein and cell binding. The biotin groups on the PEG chains allow for specific binding of NeutrAvidin (Thermo Fisher Scientific, Waltham, MA, USA), which in turn binds to biotinylated ligands of interest, enabling modular and specific patterning of functional components on the SLB surface. The lipids were then resuspended in 2 mL of deionized water directly by pipetting. The final lipid concentration was 0.5 mg/mL. The solution was kept chilled on ice, and a probe-tip sonicator (Sonics Vibra Cell, Sonics & Material, Newton, CT, USA) was used to generate small unilamellar vesicles (SUVs). Solid debris from the sonication process was removed by centrifuging the mixture at 20,000× *g* and 4 °C for 4 h, and the supernatant containing the SUVs was transferred to a fresh tube. All lipid vesicles were stored at 4 °C until further use. Glass coverslips, used as supported bilayer substrates, were cleaned by sonication in a 1:1 mixture of isopropanol and water for 30 min. After extensive rinsing with water, coverslips were further cleaned by incubating in 50% H_2_SO_4_ overnight, followed by UV treatment in an enclosed UV ozone generator (UV/Ozone ProCleaner Plus, Ames, IA, USA) for 30 min. The glass coverslips were then washed thoroughly with water and dried under an N_2_ stream. The self-assembly of lipid bilayers was performed by adding a 1:1 mixture of lipid vesicles and 2× Tris-buffered saline (TBS; Sigma-Aldrich, St. Louis, MO, USA) to the coverslips and incubating for 5 min at room temperature. These bilayer-supporting coverslips were each assembled underwater to avoid contact with the external atmosphere for further experiments.

### 4.4. Micropatterned Supported Lipid Bilayer Preparation

Micropatterned SLBs were prepared on UV-patterned polymer-coated glass substrates [31,34,35,36,37,44,65,66,67,68]. The acid cleaned and UV treated glass coverslips were dried and incubated with poly(L-lysine)-graft-poly (ethylene glycol)-biotin (PLL-*g*-PEG) (PLL (20 kDa)-*g*-[3.4]-PEG(2 kDa)) PLL-*g*-PEG-biotin (Nanocs Inc., New York, NY, USA) at a concentration of 1 mg/mL for 2 h, rinsed and dried under N_2_ stream. Micropatterns were created on the substrates by deep UV etching of the PLL-*g*-PEG-biotin polymer coating using a photomask (Bonda Technology Pte Ltd., Singapore) containing transparent circular patterns of 2 μm diameter and 4 μm spacing in all directions for 8 min. Substrates were then rinsed extensively with water to remove any residual polymer and dried under the N_2_ stream. Bilayers were then assembled on the micropatterned substrates as described above.

### 4.5. Functionalization of SLB with S1-RBD

SLB substrates assembled as described above were rinsed thrice with 5 mL 1× Tris-Buffered Saline (TBS) and incubated with 0.01% Bovine Serum Albumin (BSA) in 1× TBS for 30 min to block non-specific binding. The SLB substrates were washed thrice with 5 mL 1× TBS and incubated with Neutravidin (5 nM) [37] (Invitrogen, Waltham, MA, USA; 1 mg/mL) for 30 min. SLBs substrates, either planar or micropatterned, were then washed thrice with 5 mL 1× TBS and incubated with 15 nM recombinantly purified, His_12_-tagged WT, Omicron, and Omicron Revertant S1-RBD proteins for 90 min at room temperature for functionalizing SLBs with the proteins via the His_12_-tag-Ni-NTA interaction. This is followed by rinsing with 15 mL 1× TBS to remove unbound protein from the SLB substrates. After another 30 min of incubation, the SLB substrates were rinsed thrice with 5 mL of imaging buffer (50 mM HEPES, 150 mM NaCl, 5 mM KCl, 1 mM MgCl_2_, 11.1 mM D-glucose, 2 mM CaCl_2_, pH 7.4) to remove any desorbed protein molecules from the SLB substrates. All incubations were performed at room temperature.

### 4.6. Cell Culture and Preparation of Cells for Live Cell Experiments

A549 cells were seeded at low densities to maintain single cells and transfected with a plasmid expressing human ACE2 with a mScarlet fluorescent protein (ACE2-mSca) in Roswell Park Memorial Institute 1680 (RPMI 1680) media supplemented with 10% FBS and 1% penicillin streptomycin, at 37 °C and in an atmosphere of 5% CO_2_. To prepare the cells for experiments, cells were washed with Dulbecco’s Phosphate-Buffered Saline (DPBS) and then detached from the surface by incubating with an enzyme-free dissociation buffer (Cell Stripper, Life Technologies, Carlsbad, CA, USA) for 10–20 min at 37 °C. Cells were pipetted gently to produce a single-cell suspension, which was then centrifuged and resuspended in the imaging buffer. Cells were incubated for 10–15 min in the imaging buffer before seeding on the bilayers. A similar approach was taken for transfecting cells with Early Endosome Antigen 1 (EEA1-RFP) for monitoring the endocytic protein dynamics.

### 4.7. Assay for Monitoring the ACE2-Mediated Spike Interaction and Internalization on Bilayer

A549 cells were processed into a single-cell suspension using cell dissociation media. The cells were pre-treated with inhibitors such as pitstop 2 (50 μM), a clathrin-mediated endocytic inhibitor, or myosin inhibitor blebbistatin (50 μM) for 10–15 min. The cells were seeded on planar or micropatterned bilayer functionalized with spike protein and incubated at 37 °C and were monitored for 1 h and imaged by Reflection Interference Contrast Microscopy (RICM) and either Total Internal Reflection Fluorescence (TIRF) or confocal microscopy to monitor the assembly of ACE2-S1-RBD clusters at the cell-SLB interface. Post incubation, cells were washed with 15 mL of chilled DPBS to remove unbound cells and then fixed with 4% paraformaldehyde for 15 min at room temperature and imaged further for quantification.

### 4.8. Monitoring the Effect of Inhibiting Endocytosis and Actin in Spike Internalization

A549 cells were either transfected with AP2U2-RFP [70], adapter protein in clathrin-mediated endocytosis or Lifeact-mKate2 [71] for monitoring the actin cytoskeleton. Pitstop 2, a known inhibitor of AP2U2, and blebbistatin, a myosin inhibitor, adversely affect the actin cytoskeleton. The cells were fixed and imaged to investigate the impact of the inhibitors on the Omicron S1-RBD uptake by determining the fraction of cells involved in the depletion of the spike functionalized corrals in comparison to the control.

### 4.9. Microscopy and Image Analysis

Imaging was performed using a Nikon Eclipse Ti inverted microscope with a 100× objective. RICM [72] images were obtained using a 530/11 nm excitation filter. The interaction of cells with the SLB substrate was visualized using RICM. TIRF-488 (green channel) used for S1-RBD and TIRF-561 (red channel) was used for imaging ACE2-mSca, EEA1-RFP, Lifeact-mKate2 [73]. All the images were analyzed with ImageJ 1.54p (NIH, Bethesda, MD, USA) or Fiji [74]. Preprocessing steps included background subtraction and noise reduction, which involved despeckling and removing outliers (radius: 1 pixel, threshold: 50). For quantitative analysis, additional processing was applied, including a Gaussian blur (radius: 1 pixel) to further refine the signal intensity. For region-specific quantification, selection tools were employed to isolate regions of interest (ROIs). The line scan function was used to measure fluorescence intensity along the planar bilayer, allowing calculation of ACE2-S1-RBD enrichment at the interface. Additionally, circular ROIs were defined around corrals to monitor the time-dependent changes in lipid and S1-RBD fluorescence (mGL) intensities. The colocalization analysis of ACE2-mScarlet (ACE2-mSca) and EEA1 with WT, Omicron, or Omicron Revertant S1-RBD was performed using colocalization tool available in Fiji [74].

### 4.10. Data Analysis and Figure Preparation

Data analysis and figure preparation GraphPad Prism (version 10) and Microsoft Excel (2016) were used for data analysis and graph preparation. Statistical significance was determined through non-parametric one-way ANOVA analysis. Figures were assembled using Adobe Illustrator 2024 (28.0.1) software.

## 5. Conclusions

In conclusion, our results indicate that the host cell-mediated interaction of Omicron S1-RBD with ACE2 likely promotes clathrin-mediated endocytosis. Additionally, the altered utilization of TMPRSS2 by Omicron has expanded viral entry into a broader range of cell types without requiring serine activation, which is reported to be essential for spike protein activation. While we have investigated ACE2 receptors in the current study, the SLB platform described here could be extended to investigate other membrane-localized receptor systems involved in SARS-CoV-2 infection. However, it is important to note certain limitations of our model system: the use of isolated, monomeric S1-RBD constructs, synthetic SLB corrals, and ACE2-overexpressing, TMPRSS2-negative A549 cells may not fully recapitulate the full-length, trimeric spike protein structure, physiological receptor densities, native viral membrane containing other proteins, or the protease environment of primary airway epithelial cells. These factors should be considered when interpreting the mechanistic insights from this study and extrapolating them into vivo settings.

## Data Availability

The original contributions presented in this study are included in the article/Supplementary Material. Further inquiries can be directed to the corresponding author.

## Supplementary Materials

The following supporting information can be downloaded at: https://www.mdpi.com/article/10.3390/ijms262010216/s1.
