# Supplementary material for: Selective Endocytosis-Mediated Omicron S1-RBD Internalization Revealed by Reconstitution of ACE2-S1-RBD Interaction on Micropatterned Membrane Substrates
Source: Int J Mol Sci. 2025 Oct 21;26(20):10216. doi: 10.3390/ijms262010216 (PMC12563039; doi:10.3390/ijms262010216)
Supplement: Supplementary file 1 [file ijms-26-10216-s001.zip › ijms-3904488-supplementary.pdf]

Supporting Information

Selective Endocytosis-mediated Omicron S1-RBD Internalization Revealed by Reconstitution of ACE2-S1-RBD Interaction on Supported Lipid Bilayer

Angelin M Philip[1], S M Nasir Uddin[1], Zeyaul Islam[2], Prasanna R. Kolatkar[2], Kabir H Biswas[1*]

Affiliations:
[1]College of Health & Life Sciences, Hamad Bin Khalifa University, Doha, Qatar
[2]Qatar Biomedical Research Institute, Doha, Qatar

ORCID:
Angelin M Philip: 0000-0001-7222-4669
Kabir H Biswas: 0000-0001-9194-4127
S M Nasir Uddin: 0009-0006-4264-2302
Zeyaul Islam: 0000-0002-5444-3910

***Correspondence:** kbiswas@hbku.edu.qa

Supplementary Text

Supplementary text 1: Plasmid sequences of pBAD33-WT-S1-RBD-mGL-His_{12}, pBAD33- Omicron-S1-RBD-mGL-His_{12} and pBAD33- Revertant-S1-RBD-mGL-His_{12}

S1-RBD   mGL-tag   12X-His

pBAD33-WT-S1-RBD-mGL-His_{12}:

ATGTTTCGCGTGCAGCCGACCGAAAGCATTGTGCGCTTTCCGAACATTACCAACCTGTGCCCGTTGGCGAAGTGTTT
AACGCGACCCGCTTTGCGAGCGTGTATGCGTGGAACCGCAAACGCATTAGCAACTGCGTGGCGGATTATAGCGTGCT
GTATAACAGCGCGAGCTTTAGCACCTTTAAATGCTATGGCGTGAGCCCGACCAAACTGAACGATCTGTGCTTTACCA
ACGTGTATGCGGATAGCTTTGTGATTCGCGGCGATGAAGTGCGCCAGATTGCGCCGGGCCAGACCGGCAAAATTGCG
GATTATAACTATAAACTGCCGGATGATTTTACCGGCTGCGTGATTGCGTGGAACAGCAACAACCTGGATAGCAAAGT
GGCGGCAACTATAACTATCTGTATCGCCTGTTTCGCAAAAGCAACCTGAAACCGTTTGAACGCGATATTAGCACCGA
AATTTATCAGGCGGGCAGCACCCCGTGCAACGGCGTGGAAGGCTTTAACTGCTATTTTCCGCTGCAGAGCTATGGCT
TTCAGCCGACCAACGGCGTGGGCTATCAGCCGTATCGCGTGGTGGTGCTGAGCTTTGAACTGCTGCATGCGCCGGCG
ACCGTGTGCGGCCCGAAAAAAAGCACCAACCTGGTGAAAAACAAATGCGTGAACGGCGGCGGCGGCAGCGGCGGCAT
GGTGAGCAAAGGCGAAGAACTGTTTACCGGCGTGGTGCCGATTCTGGTGGAACTGGATGGCGATGTGAACGGCCATA
AATTTAGCGTGCGCGGCGAAGGCGAAGGCGATGCGACCAACGGCAAACTGACCCTGAAATTTATTTGCACCACCGGC
AAACTGCCGGTGCCGTGGCCGACCCTGGTGACCACCCTGGGCTATGGCGTGGCGTGCTTTGCGCGCTATCCGGATCA
TATGAAACAGCATGATTTTTTTAAAAGCGCGATGCCGGAAGGCTATGTGCAGGAACGCACCATTAGCTTTAAAGATG
ATGGCACCTATAAAACCCGCGCGGAAGTGAAATTTGAAGGCGATACCCTGGTGAACCGCATTGTGCTGAAAGGCATT
GATTTTAAAGAAGATGGCAACATTCTGGGCCATAAACTGGAATATAACTTTAACAGCCATAAAGTGTATATTACCGC
GGATAAACAGAAAAACGGCATTAAAGCGAACTTTAAAACCCGCCATAACGTGGAAGATGGCGGCGTGCAGCTGGCGG
ATCATTATCAGCAGAACACCCCGATTGGCGATGGCCCGGTGCTGCTGCCGGATAACCATTATCTGAGCCATCAGAGC
AAACTGAGCAAAGATCCGAACGAAAAACGCGATCATATGGTGCTGAAAGAACGCGTGACCGCGGCGGGCATTACCCA
TGATATGGATGAACTGTATAAAGGCAGCGGCGGCAGCCATCATCATCATCATCATCATCATCATCATCATCATTAA

pBAD33- Omicron-S1-RBD-mGL-His_{12}:

ATGTTTCGCGTGCAGCCGACCGAAAGCATTGTGCGCTTTCCGAACATTACCAACCTGTGCCCGTTGATGAAGTGTTT
AACGCGACCCGCTTTGCGAGCGTGTATGCGTGGAACCGCAAACGCATTAGCAACTGCGTGGCGGATTATAGCGTGCT
GTATAACCTGGCGCCGTTTTTTACCTTTAAATGCTATGGCGTGAGCCCGACCAAACTGAACGATCTGTGCTTTACCA
ACGTGTATGCGGATAGCTTTGTGATTCGCGGCGATGAAGTGCGCCAGATTGCGCCGGGCCAGACCGGCAACATTGCG
GATTATAACTATAAACTGCCGGATGATTTTACCGGCTGCGTGATTGCGTGGAACAGCAACAAACTGGATAGCAAAGT
GAGCGGCAACTATAACTATCTGTATCGCCTGTTTCGCAAAAGCAACCTGAAACCGTTTGAACGCGATATTAGCACCG
AAATTTATCAGGCGGGCAACAAACCGTGCAACGGCGTGGCGGGCTTTAACTGCTATTTTCCGCTGCGCAGCTATAGC
TTTCGCCCGACCTATGGCGTGGGCCATCAGCCGTATCGCGTGGTGGTGCTGAGCTTTGAACTGCTGCATGCGCCGGC
GACCGTGTGCGGCCCGAAAAAAAGCACCAACCTGGTGAAAAACAAATGCGTGAACGGCGGCGGCGGCAGCGGCGGCA
TGGTGAGCAAAGGCGAAGAACTGTTTACCGGCGTGGTGCCGATTCTGGTGGAACTGGATGGCGATGTGAACGGCCAT
AAATTTAGCGTGCGCGGCGAAGGCGAAGGCGATGCGACCAACGGCAAACTGACCCTGAAATTTATTTGCACCACCGG
CAAACTGCCGGTGCCGTGGCCGACCCTGGTGACCACCCTGGGCTATGGCGTGGCGTGCTTTGCGCGCTATCCGGATC
ATATGAAACAGCATGATTTTTTTAAAAGCGCGATGCCGGAAGGCTATGTGCAGGAACGCACCATTAGCTTTAAAGAT
GATGGCACCTATAAAACCCGCGCGGAAGTGAAATTTGAAGGCGATACCCTGGTGAACCGCATTGTGCTGAAAGGCAT
TGATTTTAAAGAAGATGGCAACATTCTGGGCCATAAACTGGAATATAACTTTAACAGCCATAAAGTGTATATTACCG
CGGATAAACAGAAAAACGGCATTAAAGCGAACTTTAAAACCCGCCATAACGTGGAAGATGGCGGCGTGCAGCTGGCG
GATCATTATCAGCAGAACACCCCGATTGGCGATGGCCCGGTGCTGCTGCCGGATAACCATTATCTGAGCCATCAGAG
CAAACTGAGCAAAGATCCGAACGAAAAACGCGATCATATGGTGCTGAAAGAACGCGTGACCGCGGCGGGCATTACCC
ATGATATGGATGAACTGTATAAAGGCAGCGGCGGCAGCCATCATCATCATCATCATCATCATCATCATCATCATTAA

pBAD33- Revertant-S1-RBD-mGL-His_{12}:

ATGTTTCGCGTGCAGCCGACCGAAAGCATTGTGCGCTTTCCGAACATTACCAACCTGTGCCCGTTTGATGAAGTGTT
TAACGCGACCCGCTTTGCGAGCGTGTATGCGTGGAACCGCAAACGCATTAGCAACTGCGTGGCGGATTATAGCGTGC
TGTATAACCTGGCGCCGTTTTTTACCTTTAAATGCTATGGCGTGAGCCCGACCAAACTGAACGATCTGTGCTTTACC

AACGTGTATGCGGATAGCTTTGTGATTCGCGGCGATGAAGTGCGCCAGATTGCGCCGGGCCAGACCGGCAACATTGC
GGATTATAACTATAAACTGCCGGATGATTTTACCGGCTGCGTGATTGCGTGGAACAGCAACAAACTGGATAGCAAAG
TGAGCGGCAACTATAACTATCTGTATCGCCTGTTTCGCAAAAGCAACCTGAAACCGTTTGAACGCGATATTAGCACC
GAAATTTATCAGGCGGGCAACAAACCGTGCAACGGCGTGGCGGGCTTTAACTGCTATTTTCCGCTGCGCAGCTATAG
CTTTCGCCCGACCTATGGCGTGGGCCATCAGCCGTATCGCGTGGTGGTGCTGAGCTTTGAACTGCTGCATGCGCCGG
CGACCGTGTGCGGCCCGAAAAAAAGCACCAACCTGGTGAAAAACAAATGCGTGAACGGCGGCGGCGGCAGCGGCGGC
ATGGTGAGCAAAGGCGAAGAACTGTTTACCGGCGTGGTGCCGATTCTGGTGGAACTGGATGGCGATGTGAACGGCCA
TAAATTTAGCGTGCGCGGCGAAGGCGAAGGCGATGCGACCAACGGCAAACTGACCCTGAAATTTATTTGCACCACCG
GCAAACTGCCGGTGCCGTGGCCGACCCTGGTGACCACCCTGGGCTATGGCGTGGCGTGCTTTGCGCGCTATCCGGAT
CATATGAAACAGCATGATTTTTTTAAAAGCGCGATGCCGGAAGGCTATGTGCAGGAACGCACCATTAGCTTTAAAGA
TGATGGCACCTATAAAACCCGCGCGGAAGTGAAATTTGAAGGCGATACCCTGGTGAACCGCATTGTGCTGAAAGGCA
TTGATTTTAAAGAAGATGGCAACATTCTGGGCCATAAACTGGAATATAACTTTAACAGCCATAAAGTGTATATTACC
GCGGATAAACAGAAAAACGGCATTAAAGCGAACTTTAAAACCCGCCATAACGTGGAAGATGGCGGCGTGCAGCTGGC
GGATCATTATCAGCAGAACACCCCGATTGGCGATGGCCCGGTGCTGCTGCCGGATAACCATTATCTGAGCCATCAGA
GCAAACTGAGCAAAGATCCGAACGAAAAACGCGATCATATGGTGCTGAAAGAACGCGTGACCGCGGCGGGCATTACC
CATGATATGGATGAACTGTATAAAGGCAGCGGCGGCAGCCATCATCATCATCATCATCATCATCATCATCATCATTA
A

Supplementary Figure

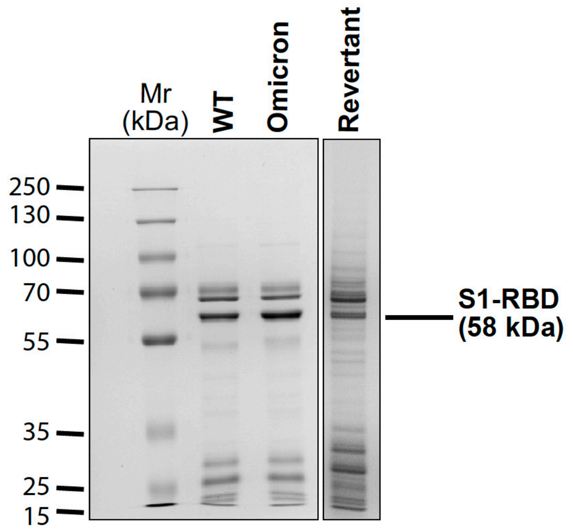

Supplementary Fig. S1. Protein expression & purification. *SDS-PAGE image showing recombinantly purified S1-RBD-mGL WT, Omicron and Omicron Revertant proteins (58 kDa). Escherichia coli BL21 (DE3) competent cells were transformed with the plasmids, pBAD33-WT-S1-RBD-mGL-His12, pBAD33-Omicron-S1-RBD-mGL-His12, or pBAD33-Omicron Revertant-S1-RBD-mGL-His, and protein expression was induced with 0.2% L-arabinose. The recombinant proteins were purified using a nickel-nitrilotriacetic acid (Ni-NTA) metal affinity chromatography.*
